# Psychological Distress and Quality of Life in Women With Endometriosis: A Narrative Review of Therapeutic Approaches and Challenges

**DOI:** 10.7759/cureus.80180

**Published:** 2025-03-06

**Authors:** Satish P Dipankar, Bhupesh Kushram, Parvathy Unnikrishnan, Jeevitha R Gowda, Ruchita Shrivastava, Arun Rajaram Daitkar, Vishal Jaiswal

**Affiliations:** 1 Department of Physiology, All India Institute of Medical Sciences, Mangalagiri, Mangalagiri, IND; 2 Department of General Surgery, Chhindwara Institute of Medical Sciences, Chhindwara, IND; 3 Gynecology and Obstetrics, Amrita School of Ayurveda, Kollam, IND; 4 Life and Allied Health, M S Ramaiah University of Applied Sciences, Bangalore, IND; 5 Department of Botany/Medicinal Plants, Society for Advanced Research in Plant Science, Narmadapuram, IND; 6 Department of Psychology, Dr. Babasaheb Ambedkar Marathwada University, Chhatrapati Sambhaji Nagar, IND; 7 Department of Anaesthesia, Banaras Hindu University, Varanasi, IND

**Keywords:** endometriosis, multidisciplinary care, psychological distress, quality of life, therapeutic approaches

## Abstract

The chronic gynecological disorder endometriosis is a debilitating condition for women’s physical and psychological health. Endometriosis is a disease that causes endometrial-like tissue to grow outside the uterus and causes debilitating symptoms, including chronic pain, fatigue, and infertility. Women with endometriosis often suffer from psychological distress, especially anxiety, depression, and social isolation, which aggravates the whole burden. The purpose of this review was to review the psychological impact of endometriosis and its influence on quality of life (QoL) and examine the relationship between physical symptomatology and mental health. It also discusses current therapeutic approaches: medical treatment, psychological interventions, and complementary therapies, and problems of delayed diagnosis, poor multidisciplinary care, and stigma. An integrated care model combining physical and psychological support is required as concluded from the review. Recommendations for future research are made, specifically in the areas of personalized care strategies and improving healthcare access to improve outcomes for women with endometriosis.

## Introduction and background

The chronic gynecological condition endometriosis impacts 10% (190 million) of women between reproductive ages across the world [1]. The condition manifests when endometrial-like tissue develops outside the uterus resulting in systemic inflammation through the activation of immune responses and cytokine release and oxidative stress [1]. The inflammatory process leads to pelvic pain as well as dysmenorrhea and infertility symptoms. The ectopic endometrial tissue most frequently develops on ovaries and fallopian tubes, peritoneum, bladder, and intestines while rare occurrences of diaphragm and lung implantations have been reported [1].

The medical community has extensively studied endometriosis's physical manifestations but has not thoroughly investigated its psychological effects [2]. The combination of persistent pain and fertility issues and delayed medical diagnosis leads numerous people to develop elevated anxiety levels depressive symptoms and increased stress. The disease affects daily functioning as well as career advancement interpersonal relationships and overall quality of life (QoL) [1,2]. Patient well-being requires a complete strategy that treats physical conditions and psychological aspects of endometriosis.

Medical progress has not solved the inadequate endometriosis care because patients still experience long diagnostic delays insufficient symptom management and restricted access to team-based medical care. Research shows diagnostic delays persist between seven and 10 years which forces numerous women to suffer with uncontrolled symptoms during this time [3]. The global survey of 1,500 endometriosis patients revealed that 60% judged their QoL to be “poor” or “very poor”, up to 50% experienced notable anxiety and 30-40% suffered from depression due to the condition [4]. The absence of consistent treatment procedures in addition to the insufficient provision of psychological resources within routine care practices intensifies the total health effects of endometriosis [5]. The research demonstrates the necessity of implementing a comprehensive healthcare model that combines gynecological care with psychological support and social assistance for better patient results [5]. Managing endometriosis is another challenge in how this deals with chronic conditions with mental health comorbidities globally. To bridge these gaps, an accessible, integrated care framework needs to be developed to take into account not only the physical but also the psychological and social aspects of the disease. Holistic, patient-centered approaches to managing the condition can not only improve the outcomes for the individual but also improve the wider socio-economic burden of this debilitating condition [6].

As the main objective of this narrative review, the authors analyze the psychological effects together with physical manifestations of endometriosis on women's QoL. The evaluation concentrates on exhibiting the diagnostic obstacles alongside treatment limitations and typically focuses on mental suffering among those affected. The study investigates how integrated care systems address physical health requirements together with mental health requirements. The assessment delves into different treatment strategies which include medical techniques alongside surgical methods, psychological treatments and alternative techniques while focusing on the need to enhance endometriosis knowledge and activist movements to eliminate the surrounding stigma

A 2023 study investigated how widespread and intense psychological distress affects women who have endometriosis. The present review analyzes published studies to detect treatment deficiencies and care complexities while exploring methods to boost patient results rather than using the statistical outcomes presented by the 2023 research. This review combines evidence about mental health effects with evaluation of treatment methods and develops an integrated care framework to treat physical and psychological symptoms. A multidomain approach delivers critical knowledge to clinical practitioners and research professionals and government officials about better patient-centered care delivery and extended disease management outcomes.

## Review

Psychological distress in endometriosis 

Women who have endometriosis develop psychological distress at higher rates, including anxiety, depression and stress-related disorders. According to research depression together with anxiety develops more frequently in women with endometriosis than in the standard population because they experience prolonged pain alongside infertility issues and diagnostic latency [6]. Data indicates that endometriosis affects half of all affected women through depressive symptoms and 30 to 40% of women show clinically significant anxiety levels (Table 1) [7]. The connection between persistent pain and mental health problems exists in both directions because persistent pain leads to elevated stress responses and hypothalamic-pituitary-adrenal (HPA) axis dysregulation and elevated inflammatory cytokines which contribute to mood disorder development [8]. The psychological strain grows stronger as the disease advances because worsening symptoms lead to deteriorating mental health results. Women who have suffered from endometriosis for extended periods or have severe cases face increased risks of developing post-traumatic stress disorder (PTSD)-like symptoms together with social withdrawal and decreased overall quality of life [9]. The existing management approaches for endometriosis focus on symptom relief instead of treating the psychological distress that has been extensively proven to accompany this condition. The need for integrated care becomes evident because it requires gynecological psychological and pain management interventions to enhance patient results [10].

**Table 1:** Prevalence of Psychological Distress in Women with Endometriosis (Source: WHO, 2023, https://www.who.int/news-room/fact-sheets/detail/endometriosis) [11]

Condition	Prevalence in Endometriosis Patients	Prevalence in General Population
Depression	50%	20-25%
Anxiety	30-40%	15-20%
Chronic Stress	30-50%	N/A
Post-Traumatic Stress Disorder (PTSD)	10-20%	5-7%

Endometriosis produces more severe emotional suffering in women who experience infertility because endometriosis stands as a primary factor behind reproductive difficulties affecting 30-50% of women with infertility [12]. The inability to conceive leads women to experience grief inadequacy feelings and psychological distress which typically worsens their depression and anxiety symptoms [12]. Social discrimination toward menstrual and reproductive health functions is a major factor that increases the emotional weight of endometriosis. Women who have endometriosis experience social isolation because of incorrect public beliefs about their condition medical dismissals and insufficient disease education in society [13]. The dismissive treatment of menstrual pain by society creates a stigma that results in delayed medical diagnosis while causing inadequate workplace and family support and limited healthcare access [4]. The mental health challenges of endometriosis become worse because women often experience self-blame and secrecy along with emotional distress [9]. The psychological weight of endometriosis drives individuals to develop unhealthy coping behaviors which reduce their psychological health and life quality [14]. Building integrated psychosocial care models in endometriosis treatment requires educational programs along with an increased understanding of these issues.

Impact on quality of life

The symptoms of endometriosis affect women by causing chronic pelvic pain in 60-80% of cases, dyspareunia in 50% of patients and severe fatigue in 70% of individuals which leads to major limitations in mobility and daily activities and work productivity [15]. The symptoms of this condition gradually intensify until patients experience both increased absence from work and diminished quality of life [16].

**Figure 1:**
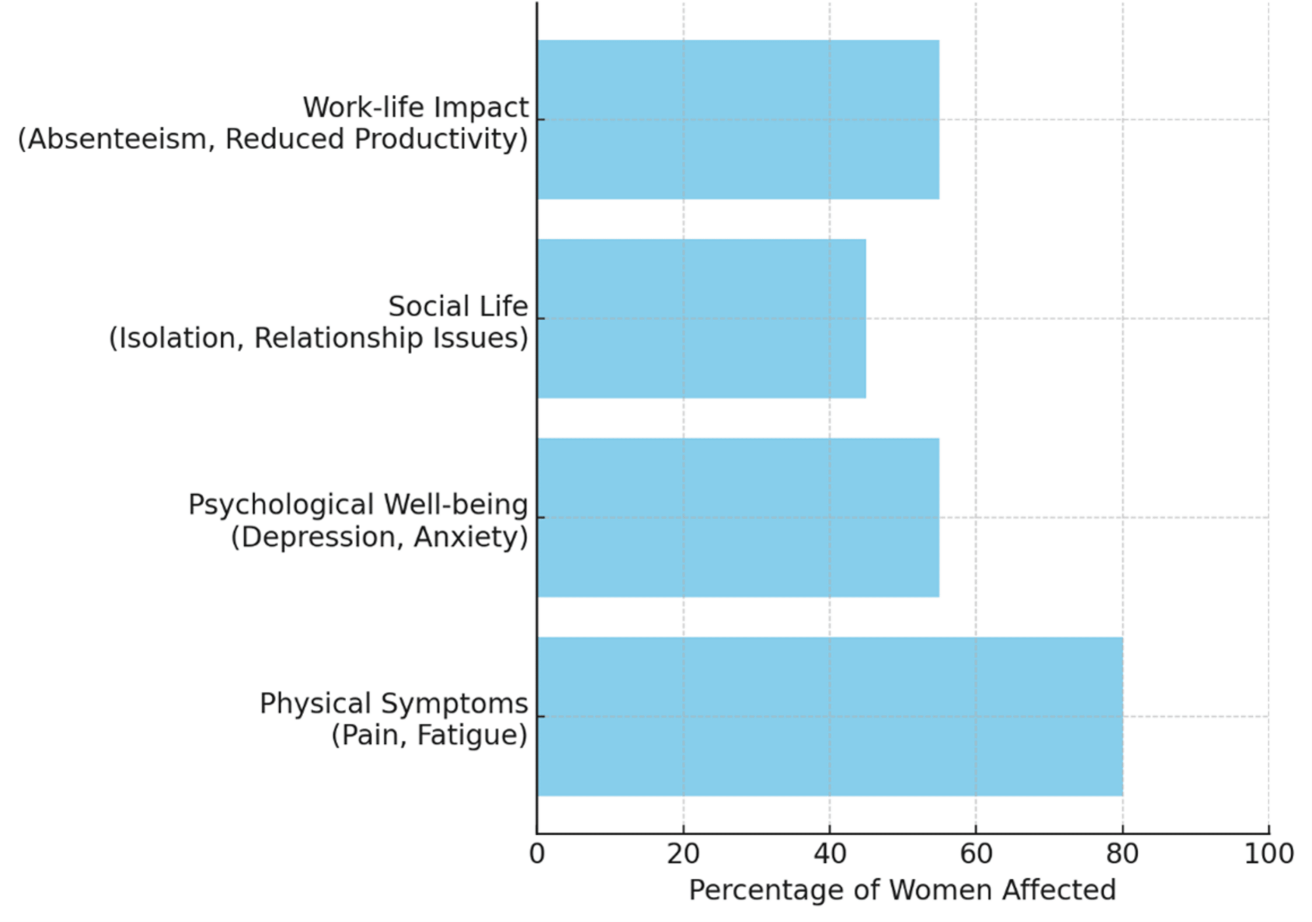
Impact of Endometriosis on Quality of Life Image created by the authors

Psychological distress affects a significant number of women with endometriosis since depression affects 30-50% and anxiety affects up to 40% of these patients as shown in Figure 1 [17]. The condition causes social isolation because patients face medical dismissal and delayed diagnosis (seven to 10 years) and menstrual health stigma [14]. Women who experience infertility problems (affecting up to 50% of cases) develop additional emotional stress which frequently affects their self-image and their relationships with others [17].

Mental resilience is paid with a toll that moves the chronic distress in a cyclical motion, where emotional and social challenges fit with each other [18]. Endometriosis is a challenge to the workplace. Frequent absenteeism, reduced productivity and difficulty maintaining a regular working schedule can result from fatigue, chronic pain, and unpredictable symptoms [19]. Women with endometriosis frequently say that their condition makes it hard to get ahead at work and maintain good working relationships. It adds to psychological distress by preventing women from contributing to their professional and personal life responsibilities because of health impairment [20].

Therapeutic approaches

Medical and Psychological Management of Endometriosis

The main treatment methods for endometriosis include relievers for pain and hormones to minimize the abnormal endometrial growth together with tissue inflammation. Coping with endometriosis pain often requires non-steroidal anti-inflammatory drugs (NSAIDs) or opioids although these inflammatory drugs lead to gastrointestinal trouble and substance abuse risks [21]. The use of hormonal medications such as oral contraceptives and gonadotropin-releasing hormone (GnRH) agonists and progestins provides pain relief and slows lesion development yet they can trigger mood changes [22]. Severe cases require surgical laparoscopy procedures which show effectiveness yet patients often experience symptom return [23,24].

Medical treatments that provide symptom relief can enhance psychological well-being yet they fail to address completely the emotional distress, anxiety and depression that stem from chronic pain and infertility along with stigma [25]. Patients who learn cognitive behavioral therapy (CBT) gain skills to manage their anxiety together with mindfulness practices that involve yoga and meditation practices to cope with pain and emotions [26,27]. Support groups help members feel less isolated while offering them social support [28].

The Role of Exercise in Endometriosis

The combination of yoga along with Pilates and aerobic exercise helps lower inflammation while improving blood circulation and reducing estrogen levels which might decrease endometriosis pain [27]. Research shows that women who practice moderate exercise patterns experience lower pain intensity levels together with better mental health outcomes [27].

Table 2 summarizes the effectiveness of medical, surgical, and psychological treatments, with evidence-based data on symptom relief, recurrence rates, and psychological improvements.

**Table 2:** Effectiveness of Various Treatments for Endometriosis NSAIDs: non-steroidal anti-inflammatory drugs, GnRH: gonadotropin-releasing hormone

Treatment	Effectiveness	Recurrence Rate	Side Effects	References
NSAIDs	Pain relief (temporary)	Low	Gastrointestinal issues	[20]
Oral Contraceptives	Reduces endometrial growth and pain	Moderate	Weight gain, mood swings	[21]
GnRH Agonists	Reduces estrogen, pain relief	High	Bone density loss	[22]
Surgical Intervention	Removes lesions, pain relief	High (Temporary)	Adhesions, organ damage	[23]
Cognitive Behavioral Therapy (CBT)	Reduces psychological distress	Moderate	Minimal	[25]

Women who have endometriosis frequently use alternative treatments such as acupuncture and make lifestyle adjustments and dietary modifications and use herbal remedies to control their symptoms. Acupuncture records positive results in pain reduction and quality of life improvement because it enhances blood circulation and controls inflammation [23].

Endometriosis patients can experience benefits from four essential lifestyle modifications which include physical exercise and proper diet management together with stress reduction and quality sleep. The combination of yoga and Pilates and swimming offers low-impact exercise that foes two things: it reduces inflammatory processes and promotes circulation while releasing endorphins for pain relief [24]. Mindfulness-based stress reduction (MBSR) together with CBT demonstrates effectiveness in boosting mental health levels of patients who suffer from endometriosis [25].

Patients with endometriosis should follow a Mediterranean diet containing omega-3 fatty acids, fiber and antioxidants because this eating pattern helps control inflammation while maintaining hormonal equilibrium. Leafy greens, cruciferous vegetables, fatty fish, whole grains together with berries and green tea represent beneficial foods [26]. Researchers have found that turmeric curcumin and ginger together with Vitex agnus-castus chasteberry and resveratrol possess anti-inflammatory hormone-regulating properties beneficial for symptom management [27].

The management of endometriosis benefits from dietary changes as well as micronutrient supplements. The immune system depends on vitamin D for proper regulation while magnesium relaxes muscles and reduces pain and zinc and selenium work together to decrease oxidative stress [28]. The nervous system function and hormonal balance receive support from B vitamins B6, B12 and folate [29].

The treatment of endometriosis and its associated symptoms includes pelvic floor physiotherapy as well as probiotics and pearlescent heating pads and transcutaneous electrical nerve stimulation (TENS) devices to reduce discomfort [29]. Further research needs to establish the long-term outcomes of these approaches because they show initial positive results. The most effective strategy for enhancing physical symptoms and psychological well-being involves medical treatment together with lifestyle modifications according to research [29].

Challenges and care

The average time to diagnose endometriosis reaches seven to 10 years because patients confuse their symptoms with other conditions and do not understand the disease and believe menstrual pain is normal [30]. Women often write off their pelvic pain together with dysmenorrhea and fatigue as typical menstrual symptoms which causes them to delay medical care. Early-stage endometriosis shows symptoms that are generally mild or intermittent which makes diagnosis difficult until pain becomes severe or infertility develops or the disease advances [31].

Women who experience persistent debilitating symptoms still encounter medical dismissal because their healthcare providers frequently diagnose their pain as irritable bowel syndrome (IBS) or pelvic inflammatory disease (PID) or psychological stress [32]. The inability of endometriosis to show up on standard imaging tests makes diagnosis more difficult because laparoscopy stands as the definitive diagnostic method but doctors frequently delay its use [33]. Certain cultures practice reproductive health stigma that prevents women from seeking medical attention because they avoid discussing menstrual pain [34].

Effective treatment faces its biggest obstacle from the absence of integrated healthcare approaches between different medical specialties. Most healthcare systems fail to provide integrated care coordination for endometriosis management between gynecologists and pain specialists and mental health professionals and nutritionists [35]. People who face social differences and cultural discrimination and financial limitations cannot readily access specialist healthcare services. Many patients face insufficient insurance coverage for endometriosis treatment, which includes laparoscopic diagnosis and hormonal therapy, thus extending their period of suffering [36].

Strategies for holistic management

**Figure 2:**
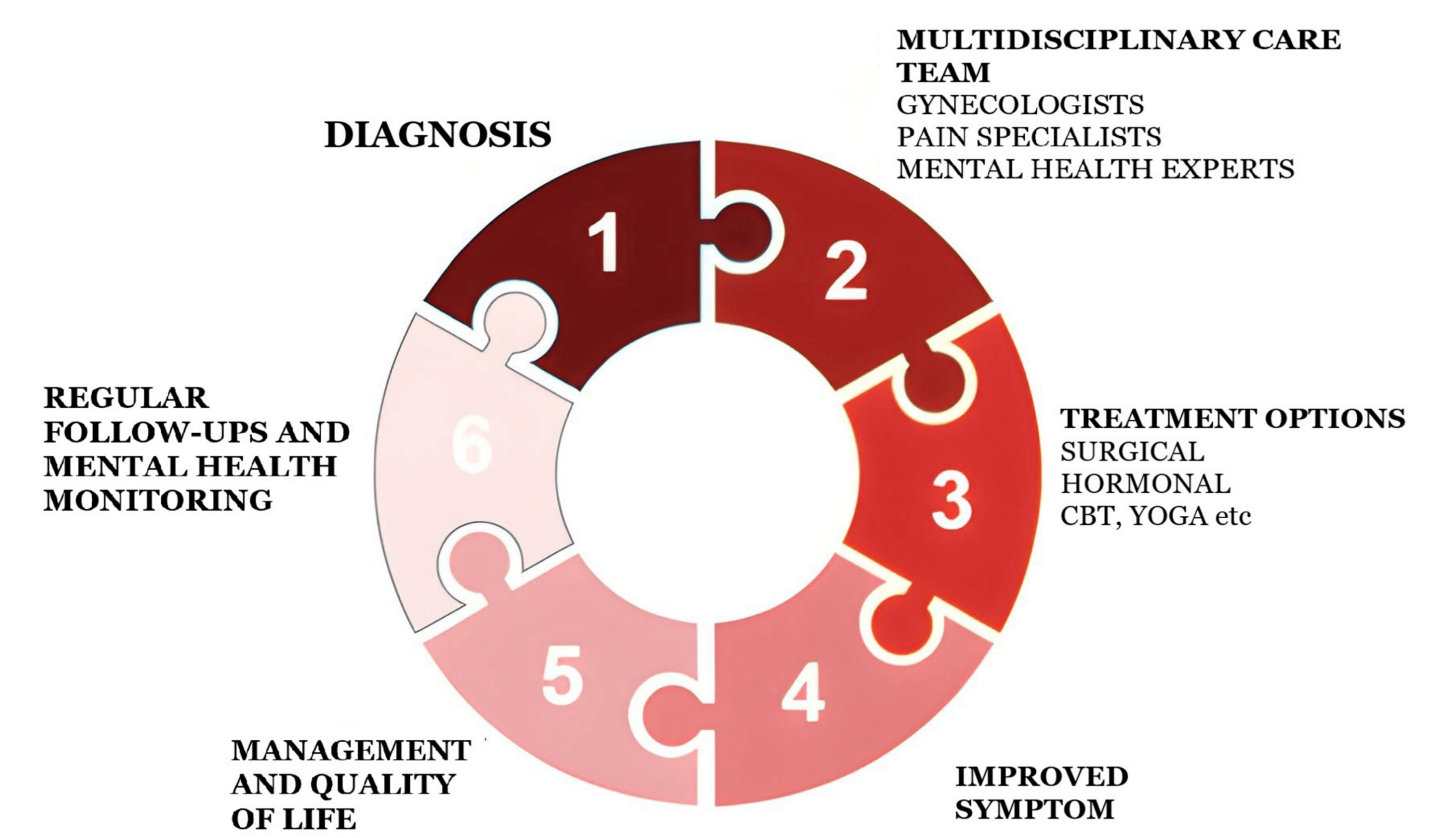
Holistic Treatment Approach for Endometriosis Image created by the authors

Blending physical and mental health care of women with endometriosis improves their quality of life. An integrated approach to treating palliative care should include both symptom management (medical and surgical interventions and other treatments) and support for psychological well-being (therapy and counseling) (Figure 2). Reducing the psychological burden, and improving the outcomes, a collaborative care model, involving specialists from multiple disciplines working together to treat the whole person, may help [37,38].

If endometriosis is to be recognized, stigma needs to be reduced and patients need to be diagnosed earlier. Future exploration needs to prioritize investigations of early identification methods, psychological stress evaluation and treatment plans, lifestyle modification methods together with healthcare service availability. Analysis of genetic and epigenetic factors would enhance risks evaluation and adjust treatment options to individual medical needs. Research must investigate the full extent of psychological challenges associated with endometriosis because it includes symptoms of depression and anxiety and PTSD-like conditions [39]. Scientific research needs to assess how well CBT and mindfulness therapies and pharmacological treatments work for treating chronic pain and mood disorders [40].

Healthcare professionals should develop multidisciplinary care models that unite gynecology services with pain management and psychological support teams. Medical research on pelvic floor therapy together with physiotherapy and hormone therapy should focus on maximizing treatment effects. The research needs to explore how anti-inflammatory diets, probiotics, herbal supplements and exercise (yoga, resistance training, aerobic activity) affect symptom relief [41].

## Conclusions

The physical health, psychological well-being and quality of life of women are significantly affected by endometriosis. Chronic pain, infertility and society's stigma of this condition place significant extra emotional burden, causing anxiety and depression. This review emphasises the necessity for holistic treatment that encompass simultaneous medical, surgical and psychological interventions to deal with physical signs and also psychological problems.

Health outcomes and quality of life of women with endometriosis can only be improved by providing psychological support as an essential part of a multidisciplinary care model with medical treatment. In addition, increasing awareness, shortening diagnostic times and decreasing access to holistic care strategies can also reduce longer-term burden of the disease. With future research, personalized care models should be developed and the value of the integrated treatment approaches should be assessed to improve both physical and mental health outcomes in affected individuals.
